# Controlling Ultrafast Magnetization Dynamics via Coherent Phonon Excitation in a Ferromagnet Monolayer

**DOI:** 10.1021/acs.nanolett.4c02325

**Published:** 2024-09-20

**Authors:** Zhaobo Zhou, Min Li, Thomas Frauenheim, Junjie He

**Affiliations:** † Department of Physical and Macromolecular Chemistry, Faculty of Science, Charles University, Prague 12843, Czech Republic; ‡ School of Science, Constructor University, Bremen 28759, Germany

**Keywords:** laser-induced coherent phonon, Fe_3_GeTe_2_, ultrafast spin dynamics, nuclei dynamics, real-time TDDFT

## Abstract

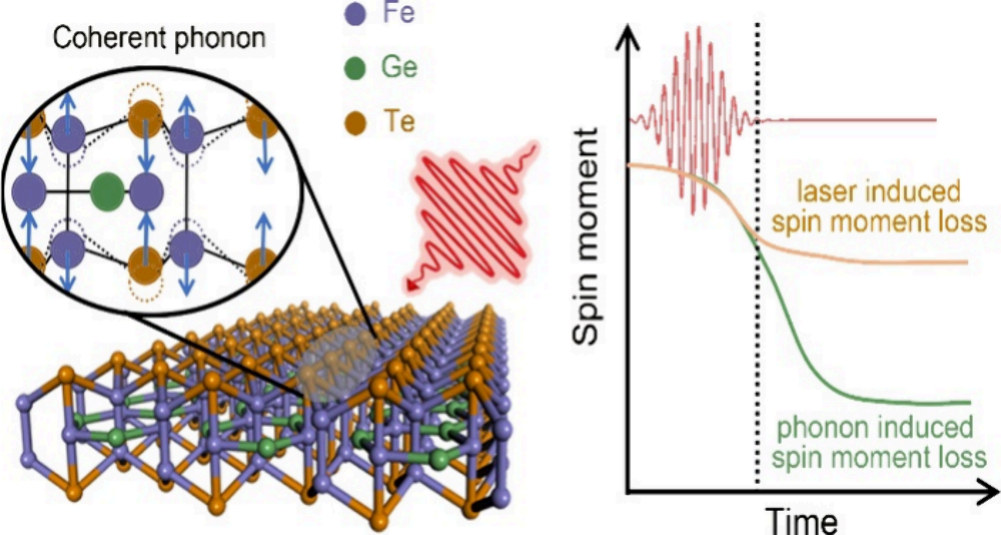

Exploring ultrafast magnetization control in 2D magnets via laser pulses is established, yet the interplay between spin dynamics and the lattice remains underexplored. Utilizing real-time time-dependent density functional theory (rt-TDDFT) coupled with Ehrenfest dynamics and nonadiabatic molecular dynamics (NAMD) simulations, we systematically investigate the laser-induced spin-nuclei dynamics with pre-excited A_1g_ and E_2g_ coherent phonons in the 2D ferromagnet Fe_3_GeTe_2_ (FGT) monolayer. Selective pre-excitation of coherent phonons under ultrafast laser irradiation significantly alters the local spin moment of FGT, consequently inducing additional spin loss attributed to the nuclear motion-induced asymmetric interatomic charge transfer. Excited spin-resolved charge undergoes a bidirectional spin-flip between spin-down and spin-up states, characterized by a subtle change in the spin moment within approximately 100 fs, followed by unidirectional spin-flip, which will further contribute to the spin moment loss of FGT within tens of picoseconds. Our results shed light on the coupling of coherent phonons with magnetization dynamics in 2D limit.

The ultrafast manipulation of magnetization dynamics via laser pulses has emerged as a focal point within the realm of opto-spintronics in recent years, owing to its rapidity and low energy consumption.^1−5^ The electromagnetic field inherent to laser pulses facilitates interactions with electronic degrees of freedom, eliciting charge excitations and indirectly coupling to spin,^6^ thereby enabling regulation of magnetization dynamics. Nevertheless, the pivotal role played by laser-induced lattice vibrations in governing ultrafast magnetization dynamics is often underappreciated. Fundamentally, the lattice acts as a reservoir for energy and momentum, into which the angular momentum lost during demagnetization is transferred.^7,8^ This lattice-mediated mechanism can precipitate spin transfers between magnetic sublattices on an ultrafast time scale,^9,10^ or potentiate exchange interactions over a longer time scale.^11^ Thus, how the lattice degree of freedom comes into play holds paramount significance for the optical manipulation of magnetization dynamics.

Coherent phonons are the quantized in-phase lattice vibrations in solids. Different from incoherent phonon, the atomic motion of coherent phonon is phase-locked across the entire photoexcited area and the atoms move back and forth in perfect synchrony (Figure S1 in the Supporting Information).^12−15^ Such a remarkable phenomenon makes the coherent phonon a fundamental medium to be used in various quantum systems (e.g., quantum dots/wells, nanolayers, superlattices) to modulate optical,^16^ electronic,^17^ magnetic,^18^ and plasmonic^19^ properties over ultrafast time scales. Recently, the concept of phonomagnetism has garnered attention, wherein light is employed to manipulate the spin degree of freedom of magnetic ions by exciting coherent phonons. Diverging from traditional optomagnetism, phonomagnetism entails the transfer of angular momentum to magnetic ions^20−22^ or the transient modulation of the lattice into a modified magnetic order.^23,24^ This concept suggests a potential avenue for directly controlling magnetization dynamics via pre-excitation of coherent phonons preceding laser-induced spin dynamics. Various experimental methodologies, such as strain pulse,^25,26^ impulsive stimulated Raman scatting,^27^ and displacive excitation of coherent phonons,^28^ have been developed to produce coherent phonons. The excitation of coherent phonons has also been instrumental in manipulating and probing light-matter interactions, leading to the discovery of novel optical and physical phenomena.^26,29−33^ However, theoretical investigations into utilizing coherent phonons for the ultrafast control of magnetization dynamics in magnetic materials, particularly in the context of two-dimensional (2D) magnets, remain scarce. Moreover, the underlying physical mechanisms governing coherent phonon-dependent magnetization dynamics processes remain ambiguous. Addressing this gap represents an urgent imperative, albeit it poses a significant challenge until two fundamental prerequisites are met. First, identifying suitable materials exhibiting Raman-active coherent lattice vibrations that can be readily excited by laser pumps is crucial. Second, developing robust theoretical frameworks capable of effectively simulating the coupling between charge/spin dynamics and atomic nuclei motion is essential.

The treatment of the spin and charge dynamics for extended solid systems via real-time, time-dependent density functional theory (rt-TDDFT) is now becoming a well-established theoretical framework. Notably, Dewhurst et al. utilized rt-TDDFT to introduce the optical intersite spin transfer (OISTR) effect, demonstrating that laser pulses can efficiently and coherently redistribute spins between distinct magnetic sublattices in magnetic materials.^9,34^ Several experimental results have also confirmed the OISTR effect in various magnetic systems.^5,10,35^ Employing rt-TDDFT, we also systematically reported the laser pulse-induced spin dynamics in 2D magnets and van der Waals heterostructures.^36−40^ However, these works primarily focus on the pure electron dynamics triggered by laser pulses, neglecting the influence of the nuclei. The coupling of the spin dynamics with nuclei poses a challenge in extended solids and remains a less-explored aspect of rt-TDDFT.^14^

In this work, using rt-TDDFT coupled with Ehrenfest dynamics and frozen phonon nonadiabatic molecular dynamics (NAMD) simulation, we systematically investigate laser-induced spin-nuclei dynamics with pre-excitation of Raman active out-of-plane A_1g_ and in-plane E_2g_ coherent phonons in the 2D ferromagnet Fe_3_GeTe_2_ (FGT) monolayer. Our simulations reveal that pre-excitation of coherent phonons leads to an additional loss of spin moment in FGT. This phenomenon can be attributed to the nuclei motion of the coherent phonon, which induced asymmetric interatomic charge transfer. After the laser pulse ceases, the magnetization dynamics of FGT exhibit two stages: spin moment remains steady for FGT with in-plane E_2g_ mode, but slightly decreases for FGT with out-of-plane A_1g_ modes within 100 fs, followed by a continuous decrease over tens of picoseconds. This behavior is attributed to an uneven spin-flip rate between the spin-up and spin-down states.

To elucidate the impact of pre-excited coherent phonons on magnetization dynamics, we initially characterize the phonon spectra of the FGT monolayer (see the computational details in the Supporting Information), as depicted in Figure 1. Previous works reported that Raman active two A_1g_ and one E_2g_ coherent phonons at Γ point in FGT few-layer or flakes are observed at room temperature.^41−43^ With a reduction in the number of layers, these Raman peaks typically undergo an upshift. Such observed phonon modes in previous experiments are also obtained in our calculated phonon spectra (labeled as cyan stars in Figure 1a), which are well in agreement with that in previous work,^44^ as shown in the inset panel in Figure 1a. Additionally, the double-pump method has been implemented in experiments where the coherent phonons can be excited by the first optical laser pump, followed by the application of the second optical laser pulse to manipulate the spin dynamics in the presence of the excited phonon modes. Herein, the generated coherent phonon in the first step is defined as the pre-excited coherent phonon. To theoretically assess the feasibility of exciting coherent phonons, we analyze the atomic displacement of each atom in the FGT monolayer under the irradiation of an out-of-plane linearly polarized pulse with a photon energy of 1.55 eV, full width at half-maximum (fwhm) of 12.21 fs and energy fluence density of 11.20 mJ/cm^2^ (see Figure S2a). It found that surface Fe (Fe1 and Fe2) and Te (Te1 and Te2) atoms exhibit out-of-plane coherent vibration, respectively, forming two out-of-plane A_1g_ phonon modes. Meanwhile, these two out-of-plane phonons are sensitive to the energy fluence density of the pulse. The atomic amplitude decreases for Fe1 and Fe2 atoms, while it increases for Te1 and Te2 atoms as the energy fluence density of the laser pulse increases to 17.50 mJ/cm^2^ (Figure S2b). This implies that the isolated out-of-plane or in-plane phonon can be excited when an appropriate laser pulse is employed. Here, three prominent coherent phonon modes, named A_1g_^1^, A_1g_^2^, and E_2g_ modes (Figure 1c-e) with the phonon amplitude set to 0.01 nm, are considered in subsequent rt-TDDFT calculations.

**Figure 1:**
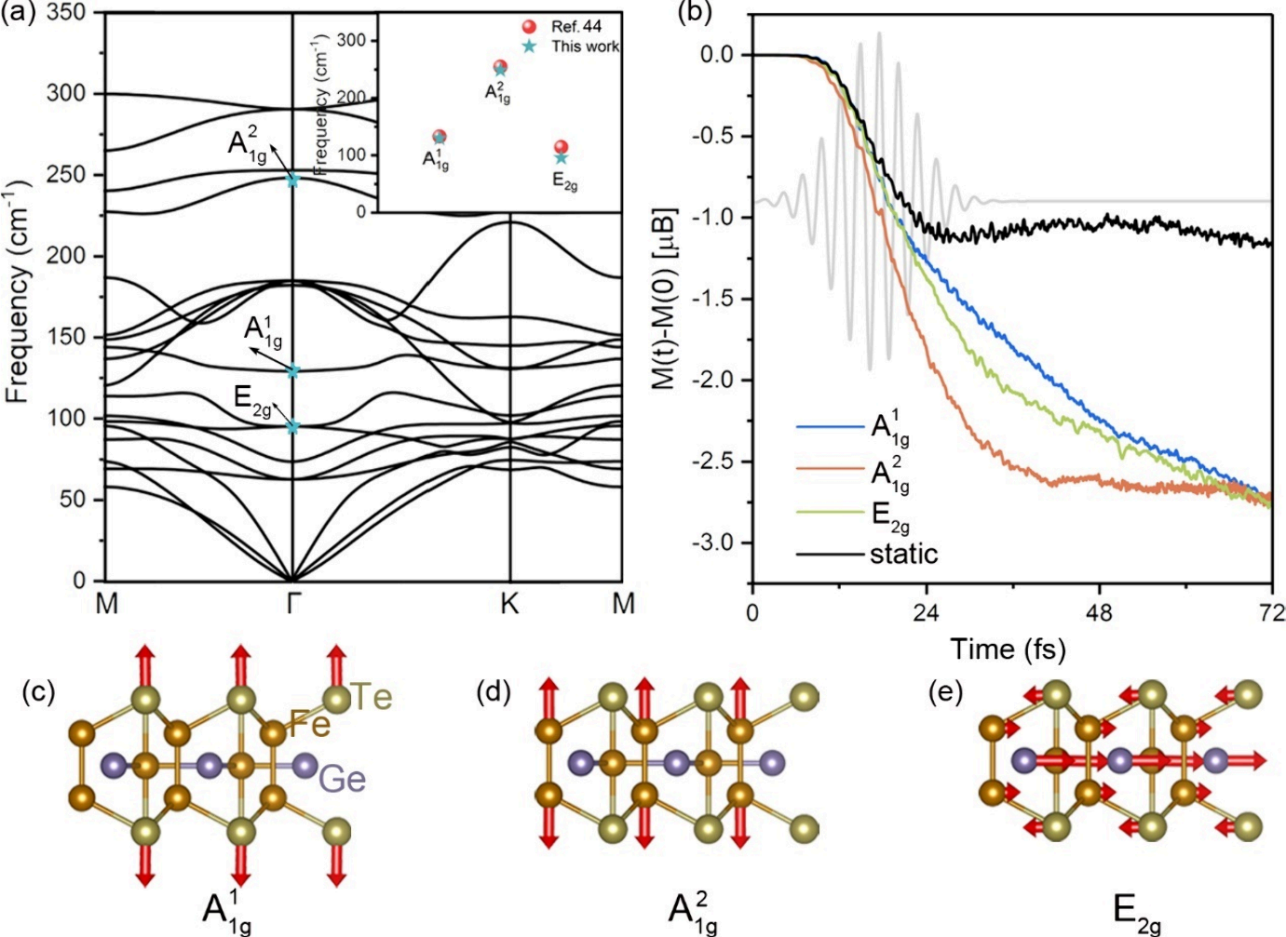
(a) Calculated phonon spectra of the FGT monolayer. The high symmetry path is the M-Γ-K-M path. The phonon frequency of two A_1g_ modes and one A_2g_ mode at Γ point are given in the inset panel, which is in agreement with the corresponding Raman shift in ref (44). (b) Change in the spin moment (Δ*M*(*t*) = *M*(*t*) – *M*(0)) dynamics of the FGT monolayer for nuclear dynamics of three coherent phonon modes (A_1g_^1^, A_1g_^2^ and E_2g_) and in the absence of nuclear dynamics (static). (c-e) Schematic diagram of two out-of-plane A_1g_ modes (A_1g_^1^ and A_1g_^2^) and the in-plane E_2g_ mode. The red arrows represent the amplitude vector.

After pre-exciting specific coherent phonons, we investigate the change in the spin moment (Δ*M*(*t*) = *M*(*t*) – *M*(0)) dynamics of the FGT monolayer in the *z*-direction under the laser pulse excitation with and without full nuclear dynamics (see the computational details in the Supporting Information), as shown in Figure 1b. It is clear that spin moment loss processes with and without nuclear dynamics demonstrate minimal discrepancy before 20 fs but exhibit additional loss with nuclear dynamics for all coherent phonons after this threshold, revealing the significant influence of coherent phonons on manipulating magnetization dynamics in FGT. This augmentation can be attributed to nuclear dynamics encompassing forces arising from both the pre-excited phonon and momentum transfer from the excited electronic system.^14^ Subsequently, as the pre-excited lattice returns to equilibrium under nuclear dynamics, the moving nuclei induce a robust back-reaction on the electronic system, e.g., nuclear motion-induced redistribution of charge density and energy level splitting. These findings underscore the pivotal role of nuclear dynamics in decisively shaping ultrafast spin moment loss, thereby facilitating expedited spin manipulation. Furthermore, the rate of spin moment loss depends on the specific pre-excited phonon type, as evidenced by the relatively faster decay in the A_1g_^2^ mode compared to that in the A_1g_^1^ and E_2g_ modes. To comprehend the origin of coherent phonon-dependent extra spin moment loss in FGT, the Δ*M*(*t*) dynamics of Fe, Ge and Te atoms, both with and without nuclear dynamics, are illustrated in Figure 2. When the nuclear motion is accounted for, a discernible decrease (increase) in the Δ*M*(*t*) of Fe (Ge/Te) atoms is observed. Especially for A_1g_^2^ mode, where Δ*M*(*t*) decreases by 1.2 Bohr magneton per Fe atom (53% of the ground-state moment) within 50 fs, surpassing the rates observed for A_1g_^1^ and E_2g_ mode, thus leading to the faster spin moment loss of FGT. However, the reverse impact of pre-excited coherent phonon-induced nuclei motion on the electronic system remains unclear. Therefore, we will delve deeper into the underlying physics of the coherent phonon-induced spin-resolved charge dynamics process in subsequent discussions.

**Figure 2:**
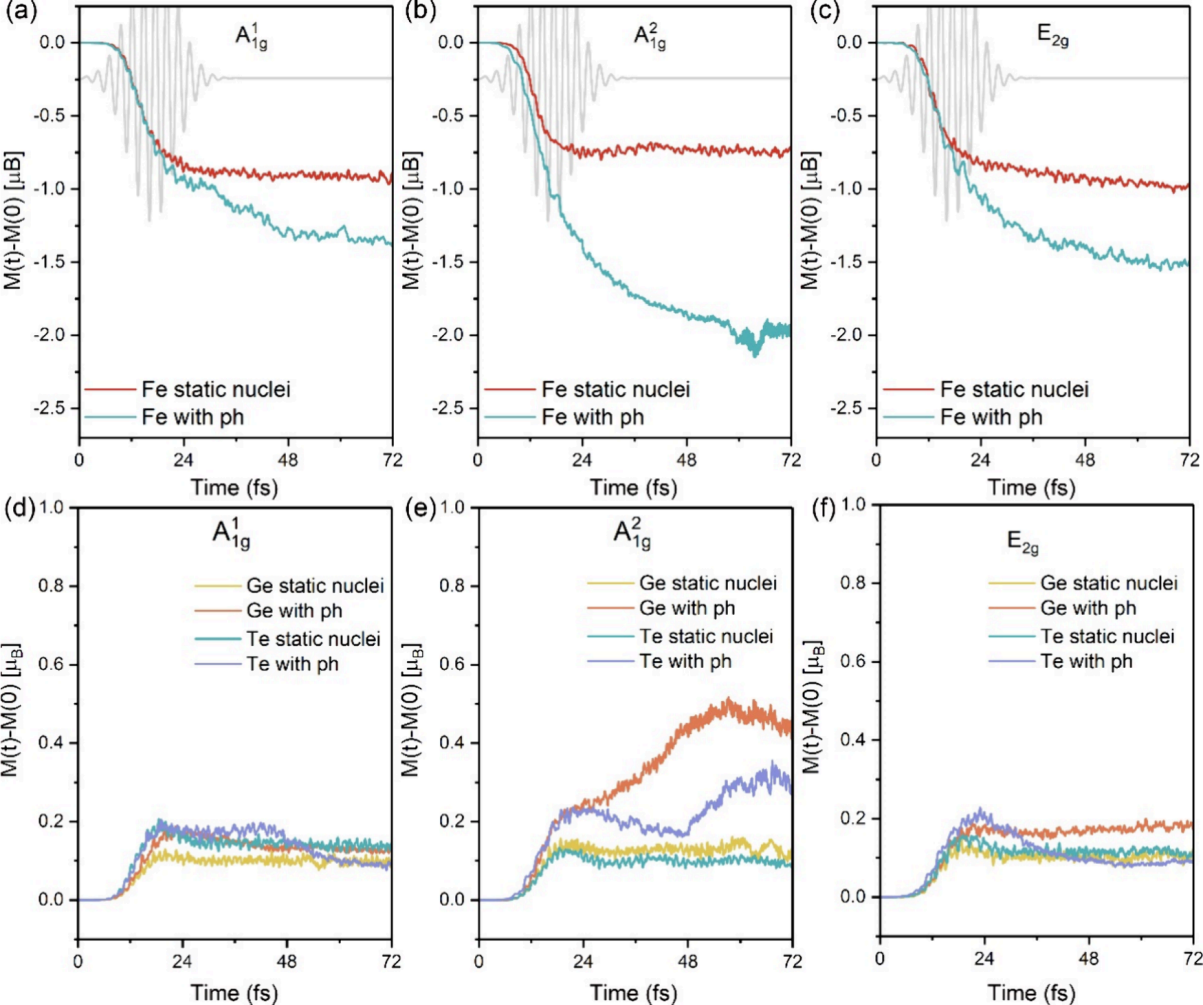
Δ*M*(*t*) dynamics of (a-c) Fe and (d-f) Ge/Te atoms in FGT for full nuclear dynamics (with ph) and the absence of nuclear dynamics (static nuclei, namely, without considering nuclear motion). Results are shown for pre-excited A_1g_^1^, A_1g_^2^, and E_2g_ coherent phonon modes.

Figure 3b-d illustrates that the time-dependent changes in spin-up charge Δ*n*_↑_ (spin-down charge Δ*n*_↓_) of Fe decrease (increase) as a function of time. The Δ*n*_↑_ (Δ*n*_↓_) shows more pronounced loss (gain) when nuclear dynamics are considered. This explains why the nuclear motion of coherent phonons amplifies the spin moment loss over a longer time scale (Figure 3a). Notably, the spin-resolved charge dynamics manifests phonon-dependent complexity. Specifically, the Δ*n*_↑_ and Δ*n*_↓_ remain relatively stable for A_1g_^1^ and E_2g_ modes, resulting in limited spin moment loss after ∼22 fs (Figure 3b,d). Conversely, for the A_1g_^2^ mode, the Δ*n*_↑_ of the Fe atom continuously decreases while the Δ*n*_↓_ increases before subsequently decreasing (Figure 3c). Such inequivalent alterations between Δ*n*_↑_ and Δ*n*_↓_ ultimately contribute to the largest spin moment loss of Fe atoms upon the A_1g_^2^ mode. To further underscore the influence of coherent phonons on magnetization dynamics, the difference between the time-dependent charge with and without nuclear dynamics (Δ*n*_ph–static_) is depicted in Figure 3e-g. As can be seen, the spin-up Δ*n*_ph-static_ exhibits a continuous and pronounced decay, while the increase in spin-down Δ*n*_ph-static_ tends to level off for E_2g_ and A_1g_^1^ modes, or even decreases in the opposite direction for A_1g_^2^ mode, indicating that the decay of spin-up Δ*n*_ph–static_ plays a predominant role in inducing substantial spin moment loss, leading to Δ*M*_A_1g_^2^_ > Δ*M*_E_2g__ > Δ*M*_A_1g_^1^_.

**Figure 3:**
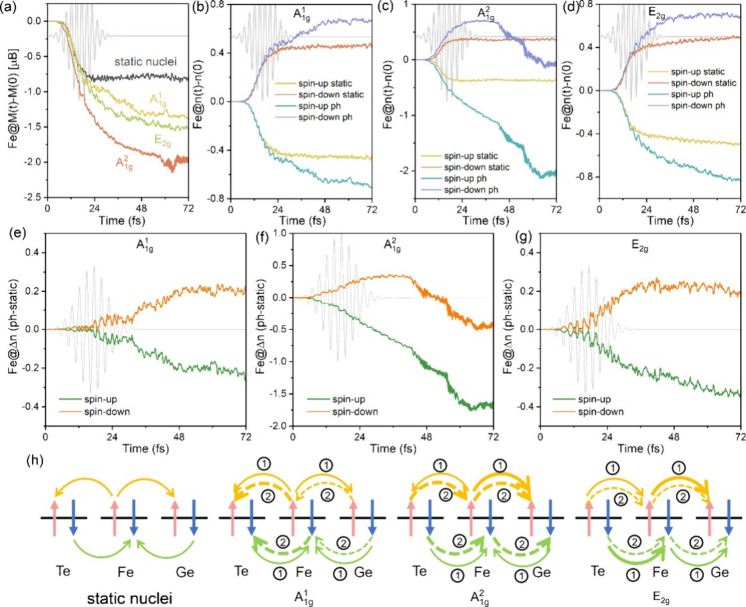
(a) Δ*M*(*t*) dynamics of Fe atom in FGT for full nuclear dynamics with pre-excited A_1g_^1^, A_1g_^2^, and E_2g_ coherent phonons and in the absence of nuclear dynamics without pre-excited coherent phonons. (b-d) Change in the spin-resolved charge (Δ*n*(*t*) = *n*(*t*) – *n*(0)) of Fe atom in FGT with (static) and without (ph) nuclear dynamics. Results are shown for pre-excited A_1g_^1^, A_1g_^2^, and E_2g_ coherent phonons, respectively. The positive (negative) value means the increase (decrease) of electrons. (e-g) Spin-resolved charge difference of Fe atom between full nuclear dynamics and in the absence of nuclear dynamics with pre-excited A_1g_^1^, A_1g_^2^, and E_2g_ coherent phonons, respectively. The positive (negative) value means the increase (decrease) of charge. (h) Schematic diagram of spin-resolved charge transfer dynamics for pre-excited A_1g_^1^, A_1g_^2^, and E_2g_ coherent phonons with and without nuclear dynamics. The dash and dot arrows represent the charge transfer dynamics pathway dominated by laser and phonon, respectively. Two processes ① and ② represent the OISTR pathway and phonon-assisted charge transfer pathway. The thicker the arrow, the larger the amount of charge transfer.

To give a clear physical description of the coherent phonon-induced spin-resolved charge dynamics process, we also examine the spin-resolved Δ*n*(*t*) of Ge and Te atoms, as depicted in Figures S3–S5. The number of spin-up and spin-down charges on Ge and Te atoms varies almost equally in the absence of nuclear dynamics, while it displays significant asymmetry when nuclear dynamics are taken into account. For instance, both Δ*n*_↑_ and Δ*n*_↓_ of Te atoms in FGT with A_1g_^1^, A_1g_^2^, and E_2g_ mode undergo a sharp increase after ∼48 fs. Interestingly, this change in Δ*n*(*t*) for Ge and Te atoms complements that of their adjacent Fe atom, suggesting that charge transfer between the Fe atom and the Ge/Te atom occurs. Note that the spin-flip (SF) mediated by spin–orbit coupling (SOC) always exhibits competition with charge transfer in charge redistribution.^45^ To figure out the effect of SOC on the coherent phonon-assisted charge dynamics process, we take the Fe atom in FGT with the A_1g_^2^ mode as an example and calculate the Δ*M*(*t*) dynamics for full nuclear dynamics with/without SOC and in the absence of nuclear dynamics with/without SOC, as illustrated in Figure S6. Remarkably, regardless of whether the coherent phonon is included, the spin moment loss of the Fe atom considering SOC slightly enhances only after the laser irradiation disappears (>20 fs). However, the spin moment loss remains consistent with that of Fe atoms without SOC during laser irradiation (<20 fs). These results affirm that the charge dynamic processes are primarily driven by charge transfer rather than SF in the early stage, with SOC slightly contributing to the demagnetization process in a later stage. In addition, we also estimate the Δ*M*(*t*) dynamics of FGT with coexcited two out-of-plane coherent phonons simultaneously and the effect of pulse fluence on magnetization dynamics (Figures S7–S9). More discussions are given in the Supporting Information.

Based on these findings, the specific charge dynamics pathways in FGT for pre-excited A_1g_^1^, A_1g_^2^, and E_2g_ coherent phonons with and without nuclear dynamics are illustrated in Figure 3h. The Fe atom will lose its spin-up charge to that of Ge and Te atoms while gaining spin-down charge from the adjacent Ge and Te atoms (static nuclei panel in Figure 3h), a phenomenon attributed to the OISTR effect. However, the charge dynamics pathway can be divided into two parts when the nuclear dynamics is considered. First, the charge transfer between Fe and Ge/Te atoms occurs via the OISTR (process ①), akin to scenarios without nuclear dynamics. After that, the nuclei motion of pre-excited A_1g_^1^, A_1g_^2^, and E_2g_ coherent phonons governs the charge redistribution between Fe and Ge/Te atoms, forming various coherent phonon-assisted charge transfer pathways over longer time scales (process ②).

So far, we have demonstrated the impact of pre-excited A_1g_^1^, A_1g_^2^, and E_2g_ coherent phonons on controlling magnetization dynamics before 100 fs and revealed the underlying physical picture behind the increased spin moment loss. In this stage, the pre-excited phonons always show a tiny atomic displacement (<0.1 Å) because the electromagnetic field of the laser pulse trends to first interact with electrons instead of lattice under the ultrafast irrigation of the pulse. Upon the disappearance of the pulse, the excited electron will relax and release energy to the lattice, further enhancing the amplitude of pre-excited A_1g_^1^, A_1g_^2^, and E_2g_ coherent phonons and affecting the magnetization dynamics in a longer time scale. To evaluate the effect of coherent phonons on such a dynamic process, the spin electron relaxation dynamics are calculated based on frozen phonon NAMD simulation^46^ (see the computational details in the Supporting Information), as shown in Figure 4. It is observed that the time evolution of spin-up energy states crosses frequently with spin-down energy states at the energy window of [0.3 0.8] eV for three coherent phonons (Figure 4a-c). This indicates that the SF can easily occur between these energy states. The energy relaxation of spin-down and spin-up states are calculated, as shown in Figure 4d-i. Figure 4g-i shows that the spin-up electron at 0.47 eV tends to rapidly flip to spin-down states within 47 fs, 86 and 85 fs for A_1g_^1^, A_1g_^2^ and E_2g_ modes respectively, corresponding to the process ③ in Figure 5a. Conversely, the relaxation of spin-down electrons at 0.68 eV can be divided into two stages (process ① and process ②). The initial SF from spin-down to spin-up states occurs within 125 fs, 212 fs, and 86 fs for A_1g_^1^, A_1g_^2^, and E_2g_ modes, respectively, followed by a slow flip back into spin-down states within 32 ps, 39 and 16 ps for A_1g_^1^, A_1g_^2^, and E_2g_ modes respectively (Figure 4d-f). Such a nonequilibrium relaxation between spin-up and spin-down electrons will significantly change the subsequent spin moment. To emphasize the unique effect of coherent phonons on charge dynamics, we also calculate the time evolution of the spin-resolved energy states and energy relaxation of spin-down electrons without freezing the A_1g_^1^, A_1g_^2^, and E_2g_ modes (Figure S10). The spin-down electron relaxes directly in the spin-down channel rather than switching to the spin-up channel, which is in contrast to the dynamic pathways observed when coherent phonon modes are excited. This result can be explained by the more disordered and entangled energy states of the spin-up and spin-down channels, which greatly reduces the energy state degeneracy and decreases the energy gap, thus enhancing the charge hopping rate between different spin-resolved energy levels.

**Figure 4:**
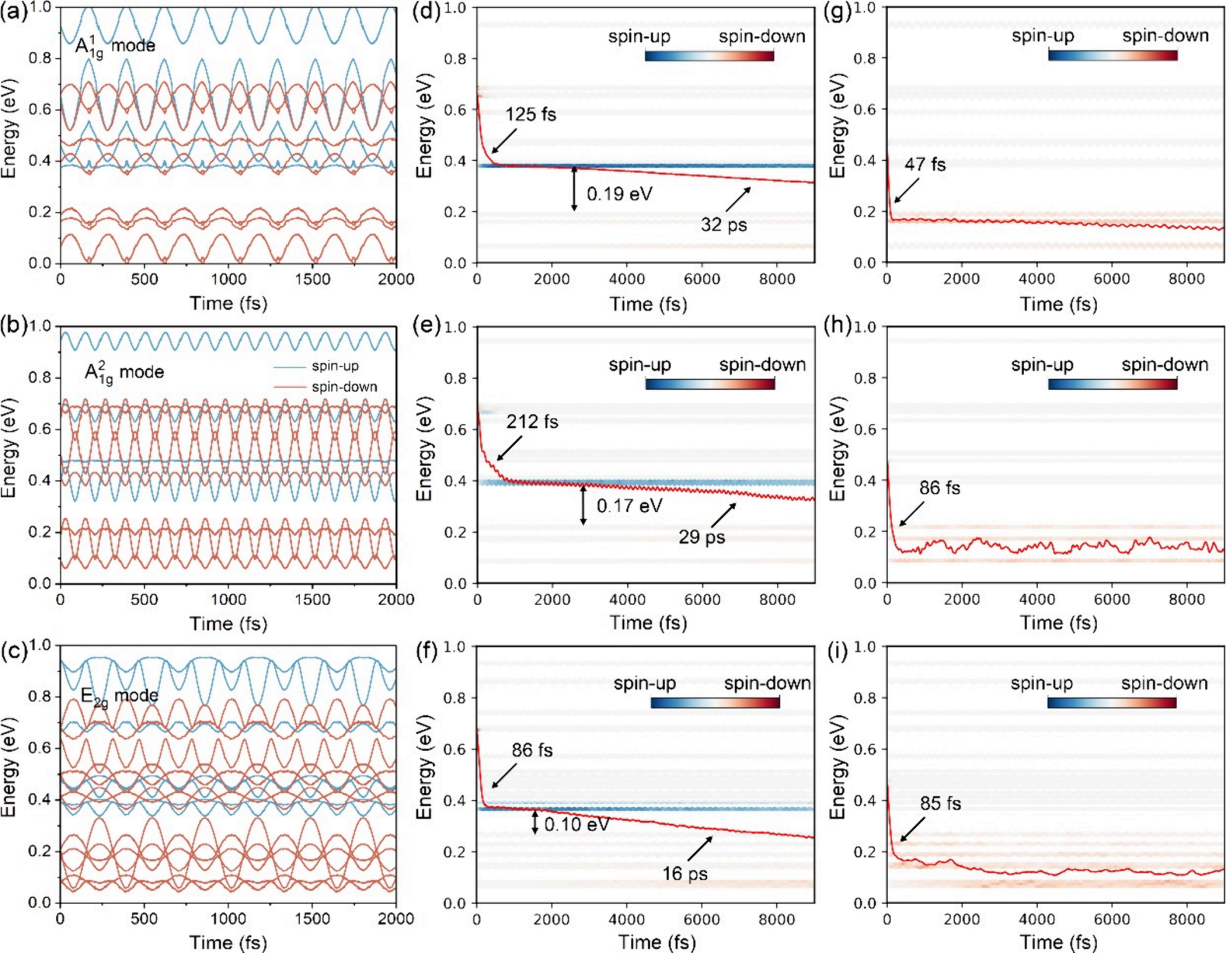
Frozen phonon NAMD simulation of spin relaxation dynamics for FGT monolayer with excited A_1g_^1^, A_1g_^2^, and E_2g_ mode at 80 K. (a-c) Time evolution of the spin-resolved energy states at the Γ point for A_1g_^1^, A_1g_^2^, and E_2g_ mode, respectively. Energy relaxation of the excited (d-f) spin-down electron and (g-i) spin-up electron for A_1g_^1^, A_1g_^2^, and E_2g_ mode, respectively. The color map indicates orbital localization. The time data are fitted using the exponential function *f*(*t*) = *a* + *b*exp(−*t*/τ).

**Figure 5:**
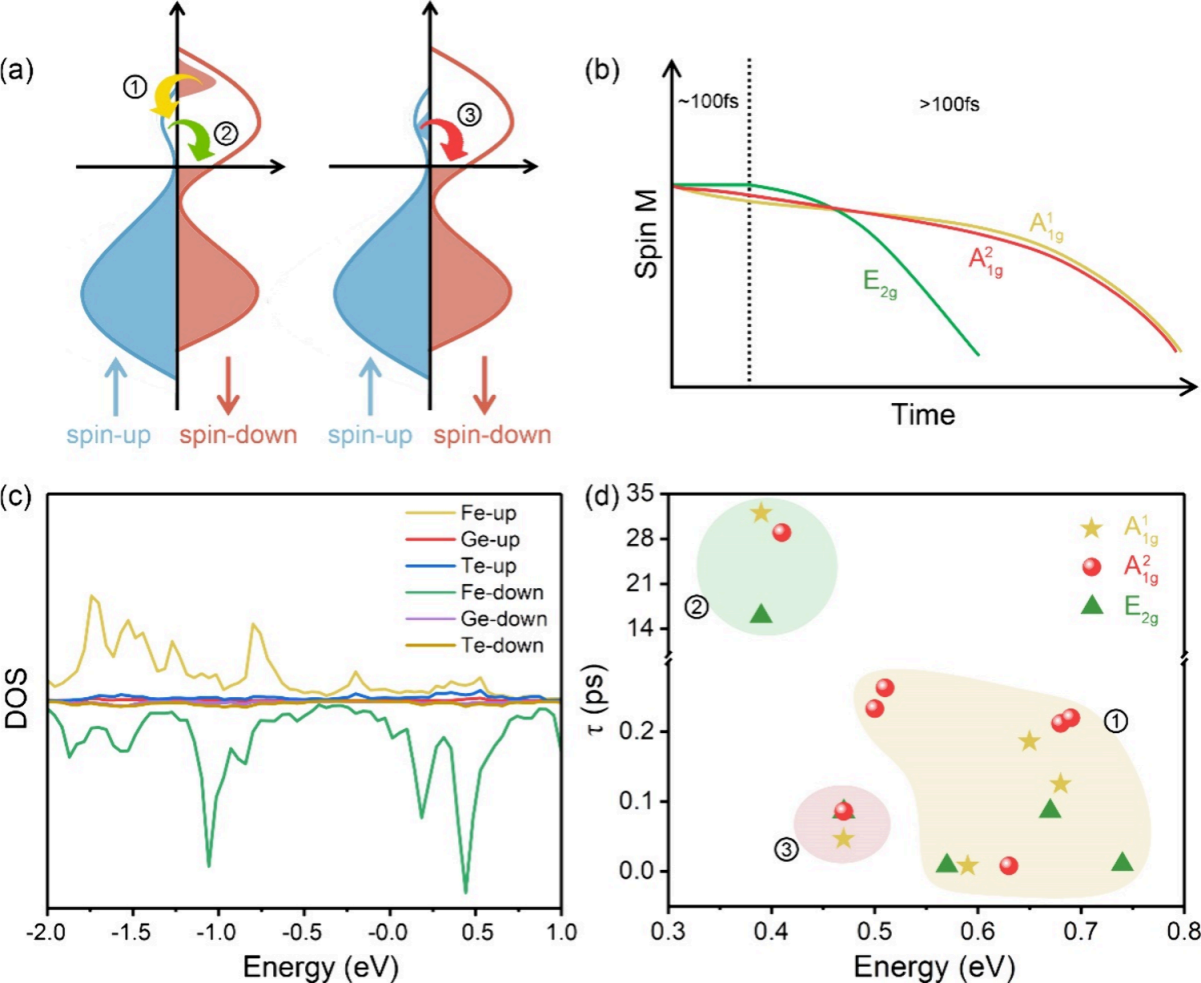
(a) Schematic diagram of spin relaxation and (b) spin loss moment in FGT monolayer with excited A_1g_^1^, A_1g_^2^, and E_2g_ mode after laser pulse excitation. Three relaxation processes are marked as process ①, process ②, and process ③, respectively. Blue and red straight arrows represent spin-up and spin-down channels, respectively. Here processes ① and ③ involve concurrent SF from the spin-down channel to the spin-up channel and from the spin-up channel to the spin-down channel, respectively, defined as bidirectional SF. While process ② only involves SF from the spin-up to spin-down channel, defined as unidirectional SF. (c) Spin-resolved DOS of each atom in the FGT monolayer. (d) Spin relaxation time of the excited spin electron. Yellow, green, and red shadows correspond to process ①, process ② and process ③. The time data are fitted using the exponential function *f*(*t*) = *a* + *b*exp(−*t*/τ).

Based on the above results, we give a clear dynamic description of spin moment loss when the laser disappears (Figure 5b). In the first stage (∼100 fs), the spin moment of FGT with two out-of-plane A_1g_ modes has a slight decrease due to the inequivalent SF rate between process ① and ③ (47 fs vs 125 fs for A_1g_^1^ and 86 fs vs 212 fs for A_1g_^2^ modes), while it remains unchanged for in-plane E_2g_ mode, attributed to the close SF rate (85 fs vs 86 fs). Then the spin moment of the in-plane E_2g_ mode starts to decrease faster than that of two out-of-plane A_1g_ modes due to the smaller SF time for the in-plane E_2g_ mode. Note that the Jahn–Teller effect is prominent in the in-plane E_2g_ mode, leading to symmetry breaking and consequently reducing the energy state degeneracy compared to the two A_1g_ modes (Figure 4a-c). This symmetry breaking further explains the observed faster spin relaxation for the E_2g_ mode. We should point out that the distribution of spin-resolved electrons excited by a laser pulse fulfills the Fermi-Direc statistics before they relax to ground states. This implies that the excited spin electron has the potential to be excited to any energy state in the window of [0.3 0.8] eV (Figure 5c). Meanwhile, some spin relaxation occurring within the same spin channel, e.g., spin-up to spin-up (up–up) or spin-down to spin-down (dn-dn) transitions, are not taken into account. This is because the spin electron maintains the initial polarization direction during up–up and dn-dn relaxation processes and thus will not impact the change in spin moment. Here, the energy relaxation involving SF for A_1g_^1^, A_1g_^2^, and E_2g_ modes at the window of [0.3 0.8] eV are shown in Figures S11–S13. The corresponding relaxation times are fitted and are shown in Figure 5b. We can see that most initial SF from spin-down states to spin-up states for A_1g_^1^ and A_1g_^2^ modes exhibit longer time compared with that for E_2g_ modes (process ①). Such SF process is slower than the SF from spin-up states to spin-down states (process ③) for A_1g_^1^ and A_1g_^2^ modes and close for E_2g_ mode, indicating that our conclusion is reasonable and universal.

In summary, we have investigated and proposed a coherent phonon-induced control strategy for the magnetization dynamics of the 2D ferromagnet FGT monolayer by performing the rt-TDDFT coupled with Ehrenfest dynamics and NAMD simulations. Our results indicate that pre-exciting coherent phonons will induce a significant change in the spin moment of Fe, Ge, and Te atoms in FGT, ultimately resulting in additional spin moment loss of the FGT monolayer. Further spin-resolved charge dynamics analysis reveals that the change in charge of Fe, Ge, and Te atoms is predominantly influenced by the nuclei motion of pre-excited coherent phonons over a longer time scale. This will lead to an asymmetric interatomic charge transfer pathway, thereby manipulating the change in the spin moment of FGT. Moreover, we explore the change in the spin moment of Fe atoms when multiple coherent phonons are simultaneously pre-excited. The results reveal that the magnetization dynamics of FGT are sensitive to the out-of-plane instead of in-plane coherent phonon. Additionally, when the laser pulse disappears, the magnetization dynamics of FGT undergoes two stages. The spin moment remains unchanged for FGT with in-plane E_2g_ mode while it slightly decreases for FGT with out-of-plane A_1g_^1^ and A_1g_^2^ modes within 100 fs. After that, the spin moment continues to decrease within tens of picoseconds. This magnetization dynamics process is attributed to an inequivalent SF rate between the spin-up and spin-down states. In light of these novel results, we suggest using a laser pulse with moderate pulse fluence to selectively pre-excite out-of-plane coherent phonons in experiments to effectively manipulate the magnetization dynamics in 2D magnets.

## Supporting Information Available

Theory methods and computational details; Atom displacement in the FGT monolayer; Change in the spin-resolved charge of Ge and Te atom; Change in spin moment of Fe atom; Snapshots of the magnetization density of FGT; Energy relaxation of the excited spin-down electron (PDF)
