# Supplementary material for: Controlling Ultrafast Magnetization Dynamics via Coherent Phonon Excitation in a Ferromagnet Monolayer
Source: Nano Lett. 2024 Sep 20;24(39):12062–9. doi: 10.1021/acs.nanolett.4c02325 (PMC11451064; doi:10.1021/acs.nanolett.4c02325)
Supplement: Supplementary file 1 — nl4c02325_si_001.pdf [file nl4c02325_si_001.pdf]

Supporting Information for

Controlling Ultrafast Magnetization Dynamics *via* Coherent Phonon Excitation in Ferromagnet Monolayer

*Zhaobo Zhou[a], Min Li[a], Thomas Frauenheim[b], Junjie He[a,]**

[a] Department of Physical and Macromolecular Chemistry, Faculty of Science, Charles University, Prague 12843, Czech Republic

[b] School of Science, Constructor University, Bremen 28759, Germany

* E-mail: junjie.he@natur.cuni.cz

Theory and methodology

For investigating the dynamics of spin and charge, we have employed state-of-the-art ab initio TDDFT methods.[1] TDDFT systematically transforms the computationally challenging problem involving electron interactions into solving the Kohn-Sham (KS) equation for noninteracting Fermions within an artificial potential. The time-dependent KS equation is:

$$i\frac{\partial\psi_j(\mathbf{r},t)}{\partial t} = \left[\frac{1}{2}\left(-i\nabla+\frac{1}{c}\mathbf{A}_{\mathrm{ext}}(t)\right)^2 + v_s(\mathbf{r},t) + \frac{1}{2c}\boldsymbol{\sigma}\cdot\mathbf{B}_s(\mathbf{r},t) + \frac{1}{4c^2}\boldsymbol{\sigma}\cdot[\nabla v_s(\mathbf{r},t)\times -i\nabla]\right]\psi_j(\mathbf{r},t) \quad (1)$$

where $\mathbf{A}_{\mathrm{ext}}(t)$ and $\boldsymbol{\sigma}$ represent the vector potential and Pauli matrices. The KS effective potential $v_s(\mathbf{r},t) = v_{ext}(\mathbf{r},t) + v_H(\mathbf{r},t) + v_{xc}(\mathbf{r},t)$ can be decomposed into the external potential v_{ext}, the classical Hartree potential v_H, and the exchange-correlation (XC) potential v_{xc}, respectively. The KS magnetic field can be written as $\mathbf{B}_s(\mathbf{r},t) = \mathbf{B_{ext}}(\mathbf{r},t)+\mathbf{B_{xc}}(\mathbf{r},t)$, where $\mathbf{B_{ext}}$ and $\mathbf{B_{xc}}$ may represent the magnetic field of the applied laser pulse plus an additional magnetic field and XC magnetic field, respectively. The last term in Eq. (1) stands for spin-orbit coupling (SOC). The magnetization density can be calculated as $\boldsymbol{m}(\boldsymbol{r},t) = \sum_j \psi_j^*(\mathbf{r},t)\boldsymbol{\sigma}\psi_j(\mathbf{r},t)$ and the integral of this vector field over the unit cell leads to the spin angular momentum.

For atom nuclear that are much heavier than electrons, their motions are treated classically on an averaged potential energy surface determined by the electronic distribution according to the Ehrenfest theorem. As the nuclei move, the effective potential from Eq. (1) will change to the following:

$$v_s(\mathbf{r},t)\rightarrow v_s(\mathbf{r},t)-\sum_{pa}\frac{\partial v_{\mathrm{cl}}(\mathbf{r})}{\partial u_\alpha^p}\delta u_\alpha^p(t) \qquad (2)$$

where p labels a nucleus and α is the direction. v_{cl} is the Coulomb potential from the nucleus and the core density of electrons. According to the Hellmann-Feynman theorem, the force acting on the nuclei can be determined, which can be calculated using rt-TDDFT. Once we know the atomic force, the displacement of the nuclei $\delta u_\alpha^p(t)$ can be calculated as follows:[2]

$$-F_{pa}(t)=-\omega\delta u_\alpha^p(t) \qquad (3)$$

Note that the nuclear motion themselves also causes a back-reaction on the electronic system, which affects the force on the nuclear again. That means it is accurate when a small displacement is applied.

The dynamics of spin-resolved charge occupation can be used to characterize the change in spin moment loss, which is expressed as follows:

$$\Delta n_\uparrow(t)=\frac{\Delta n(t)+\Delta M(t)}{2},\ \Delta n_\downarrow(t)=\frac{\Delta n(t)-\Delta M(t)}{2} \qquad (4)$$

where $\Delta n(t)=n(t)-n(t=0)$ represents the change in local charge compared to the initial charge. $\Delta n_\uparrow(t)$ and $\Delta n_\downarrow(t)$ denote the time-dependent changes in spin-up and spin-down charges, respectively. Simplifying the Eq. (4) we obtain the $\Delta M(t)=\Delta n_\uparrow(t)-\Delta n_\uparrow(t)$, namely the higher difference between $\Delta n_\uparrow(t)$ and $\Delta n_\downarrow(t)$ indicate greater spin moment loss.

Computational details

The structural optimization was implemented with the Vienna Ab initio Simulation Package (VASP).[3,4] The Perdew-Burke-Ernzerhof (PBE) functional within the generalized gradient approximation was employed to account for exchange-correlation interactions.[5] Electron-ion interaction was described using the projector-augmented wave method.[6] A cutoff energy of 500 eV and a Monkhorst-Pack 15×15×1 k-mesh grid were utilized. The lattice constants and atomic positions were fully relaxed until the atomic forces were smaller than 0.1 meV Å^{-1}. The electron relaxation convergence criterion was 10^{-7} eV. A vacuum region of more than 15 Å in the z direction was used to avoid spurious interactions with the neighboring cells. Phonon calculations were conducted using the finite difference scheme implemented in Phonopy software,[7] adopting a finite-displacement approach with a 0.01 Å displacement and a 4×4×1 supercell.

Laser-induced spin dynamics calculations were implemented with the ELK code[8] using a fully noncollinear version of rt-TDDFT. A regular mesh in a k-space of 8×8×1, a smearing width of 0.027 eV, and a time step of Δt=0.1 a.u. were used. All calculations were performed using an adiabatic local spin density approximation (ALSDA). The external optical field was defined as a linearly polarized pulse with a photon energy of 1.55 eV, full width at half maximum (FWHM) of 12.21 fs, and a pulse fluence of 11.20 mJ/cm^2. TDDFT coupled with Ehrenfest nuclear dynamics simulation involves a two-step process: First, the forces under the influence of a laser pulse are calculated. In the second step, the same calculation is repeated but this time the forces are used to find the motion of the nuclei. The back-reaction of this nuclear motion is applied to the electronic system.

The spin relaxation dynamics simulations were implemented with the Hefei-NAMD code (Hefei-NAMD_SOC version).[9,10] The nuclear degrees of freedom are treated classically and are unaffected by the dynamics of the electronic degrees of freedom, which is known as classical-path approximation (CPA).[11] To understand how the carrier dynamics depend on a specific phonon mode,

the frozen phonon method is performed. After the geometry structures were optimized using VASP, the out-of-plane A_{1g}^1, A_{1g}^2 modes and in-plane E_{2g} modes were chosen and generated the atomic vibration trajectory that the vibrational amplitude is determined by the average kinetic energy of phonon mode as $\langle K \rangle = 3N \cdot (K_B T/2)$.[12] N and T represent the number of atoms and temperature. Here we choose a reasonable T= 80K since the T_c of the FGT monolayer has been reported between 50K~130 K.[13,14] After that, 100 initial configurations were selected randomly in the phonon vibration period and 20000 NAMD trajectories were sampled for each chosen initial structure. The 225 fs, 119 fs and 325 fs nonadiabatic (NA) Hamiltonians are iterated to perform 9 ps NAMD simulation for A_{1g}^1, A_{1g}^2 and E_{2g} modes respectively.

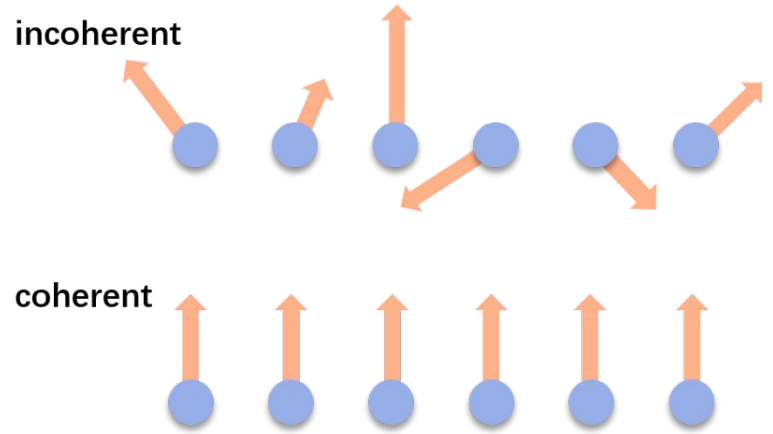

Figure S1. Schematic diagram of incoherent and coherent phonon vibration. The blue spheres and orange arrows represent the atoms and their vibration vector, respectively.

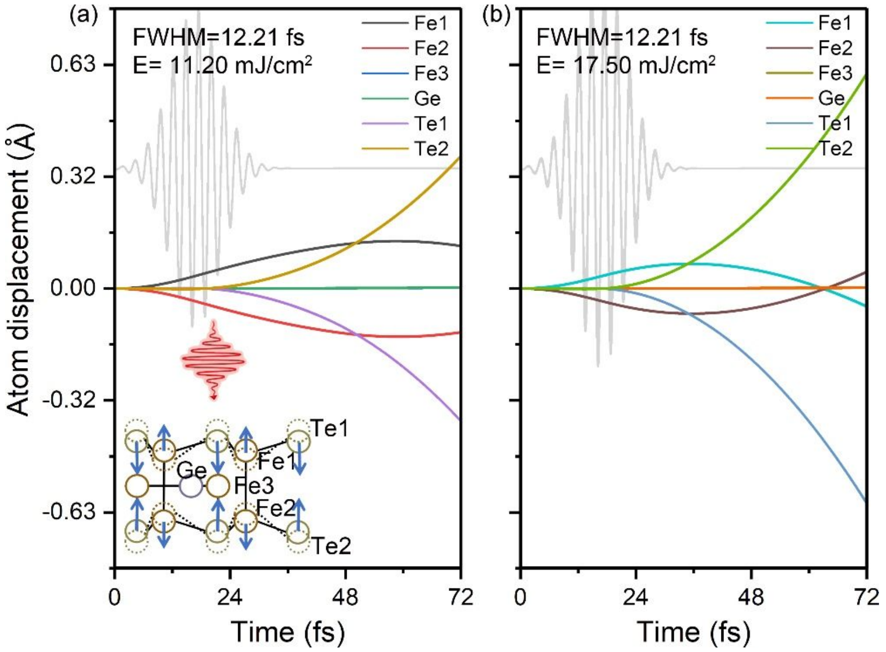

Figure S2. The atom displacement of each atom in the FGT layer under the pre-excitation of laser pulse with different energy fluence density. The linearly polarized laser pulse (grey line) with a photon energy of 1.55 eV, FWHM of 12.21 fs, fluence density of (a) 11.20 mJ/cm^2 and (b) 17.50 mJ/cm^2 along the out-of-plane direction are applied.

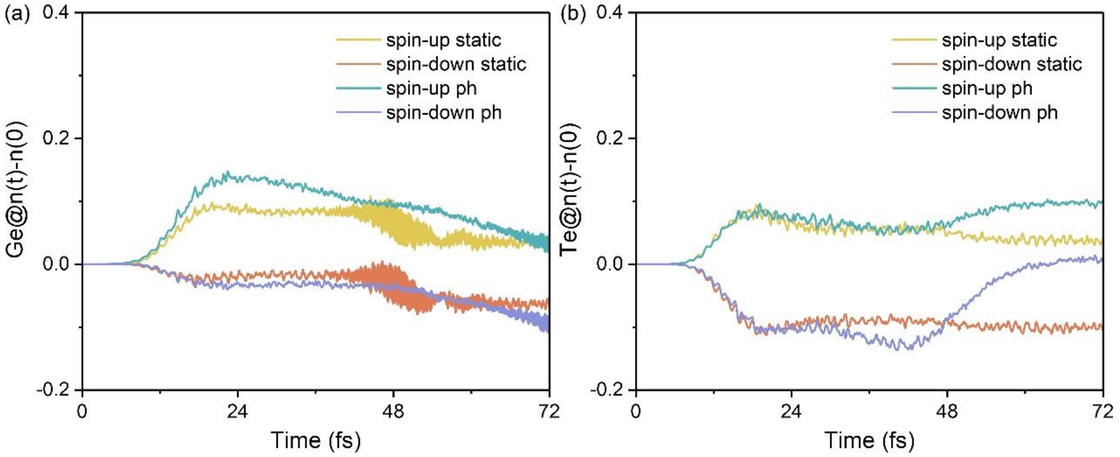

Figure S3. Change in the spin-resolved charge ($\Delta n(t) = n(t) - n(0)$) of (a) Ge and (b) Te atom in FGT with (static) and without (ph) nuclear dynamics. Results are shown for pre-excited A_{1g}^1 coherent phonons. The positive (negative) value means the increase (decrease) of electrons.

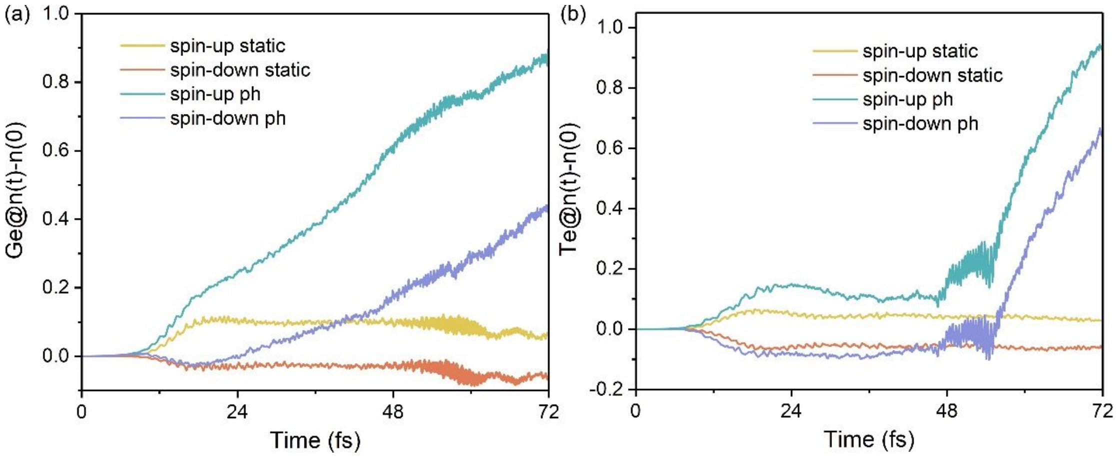

Figure S4. Change in the spin-resolved charge ($\Delta n(t) = n(t) - n(0)$) of (a) Ge and (b) Te atom in FGT with (static) and without (ph) nuclear dynamics. Results are shown for pre-excited A_{1g}^2 coherent phonons. The positive (negative) value means the increase (decrease) of electrons.

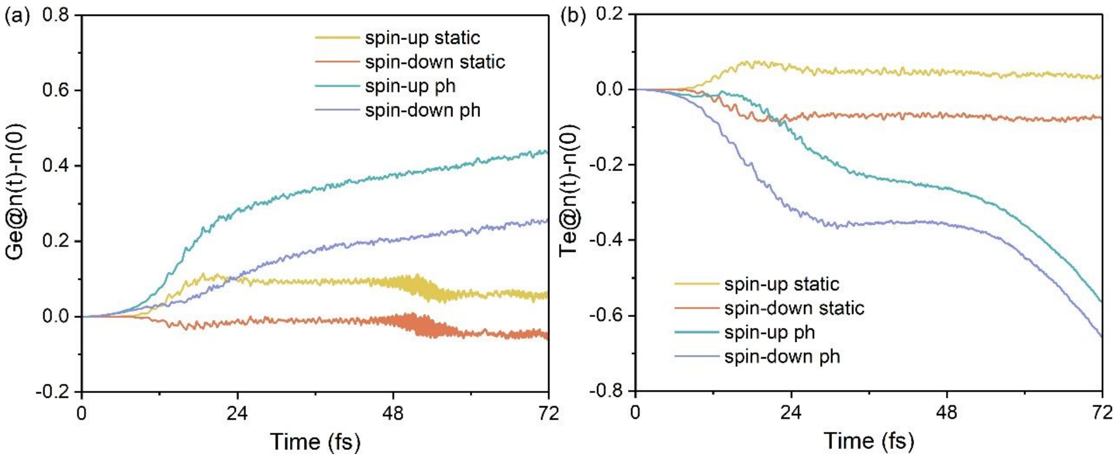

Figure S5. Change in the spin-resolved charge ($\Delta n(t) = n(t) - n(0)$) of (a) Ge and (b) Te atom in FGT with (static) and without (ph) nuclear dynamics. Results are shown for pre-excited E_{2g} coherent phonons. The positive (negative) value means the increase (decrease) of electrons.

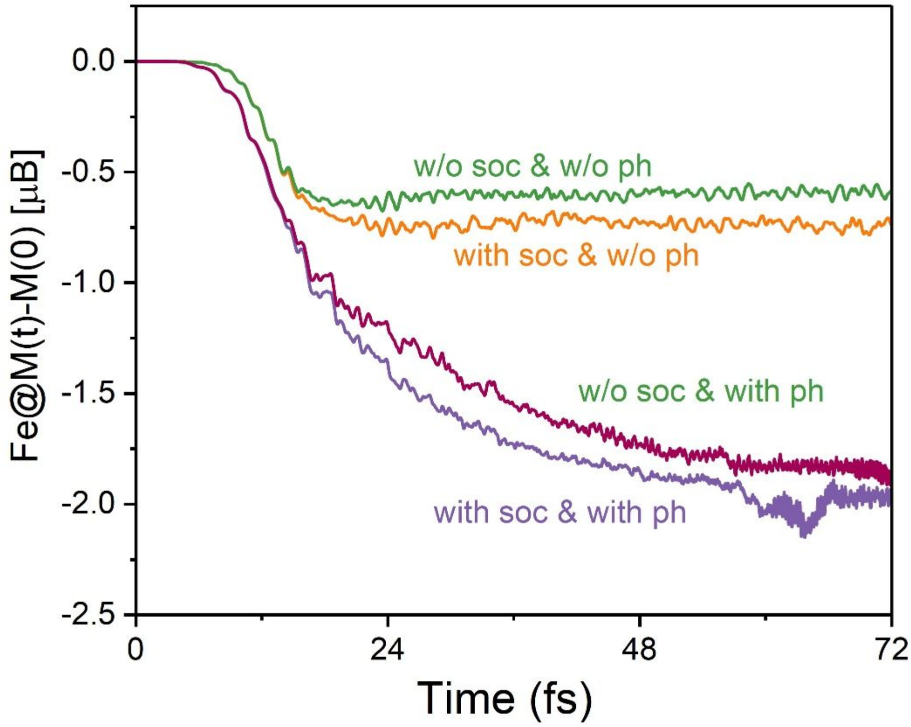

Figure S6. Change in spin moment $\Delta M(t)$ of Fe atom for full nuclear dynamics (with ph) and in the absence of nuclear dynamics (w/o ph) of pre-excited A_{1g}^2 coherent phonons. The results with and without SOC (w/o soc) are also calculated.

Phonon type-dependent spin moment loss of Fe atom

In Figure S2, we demonstrate that two out-of-plane A_{1g} modes can be simultaneously pre-excited

under the laser pulse with specific fluence density. i.e. 11.20 mJ/cm^2. Under this premise, the spin moment loss of the Fe atom is explored when out-of-plane A_{1g}^1 and A_{1g}^2 modes are excited together, as shown in Figure S7a. We observed that the spin moment loss of Fe atoms for co-excited A_{1g}^1 and A_{1g}^2 is close to that for isolated A_{1g}^2 mode, suggesting that the mode exerting the strongest influence on magnetization will predominantly govern the magnetization dynamics. In addition, the in-plane E_{2g} coherent phonon can be regarded as a collection of in-plane Fe mode, in-plane Ge mode and in-plane Te modes. Consequently, we examine the spin moment loss for these three modes respectively, as shown in Figure S7b. In contrast to the out-of-plane A_{1g}^1 and A_{1g}^2 modes, the discrepancy between these three in-plane modes is negligible. Therefore, we propose experimentally manipulating magnetization dynamics by selectively pre-exciting out-of-plane coherent phonons, as they exhibit higher sensitivity to controlling spin moment loss compared to in-plane phonons. To gain further insights into spin transfer processes, we analyze the time evolution of magnetization density in FGT with pre-excited A_{1g}^1, A_{1g}^2 and E_{2g} modes, as shown in Figure S7c and Figure S8. Notably, a larger transfer of spin moment from Fe atoms to adjacent Ge/Te atoms is observed in FGT with pre-excited A_{1g}^2 mode after ~21 fs, further demonstrating the significant influence of A_{1g}^2 mode on manipulating the magnetization dynamics of FGT. Here the multiple coherent phonon excitations with phase difference as well as non-zero amplitude vector angle, which could form chiral phonons, are not considered in this work. Some specific pulse light sources, e.g. circularly polarized light, can be employed to excite chiral phonons, potentially inducing more exotic photo-induced magnetism and optical phenomena,[15–18] and are worthy of further exploration in the future.

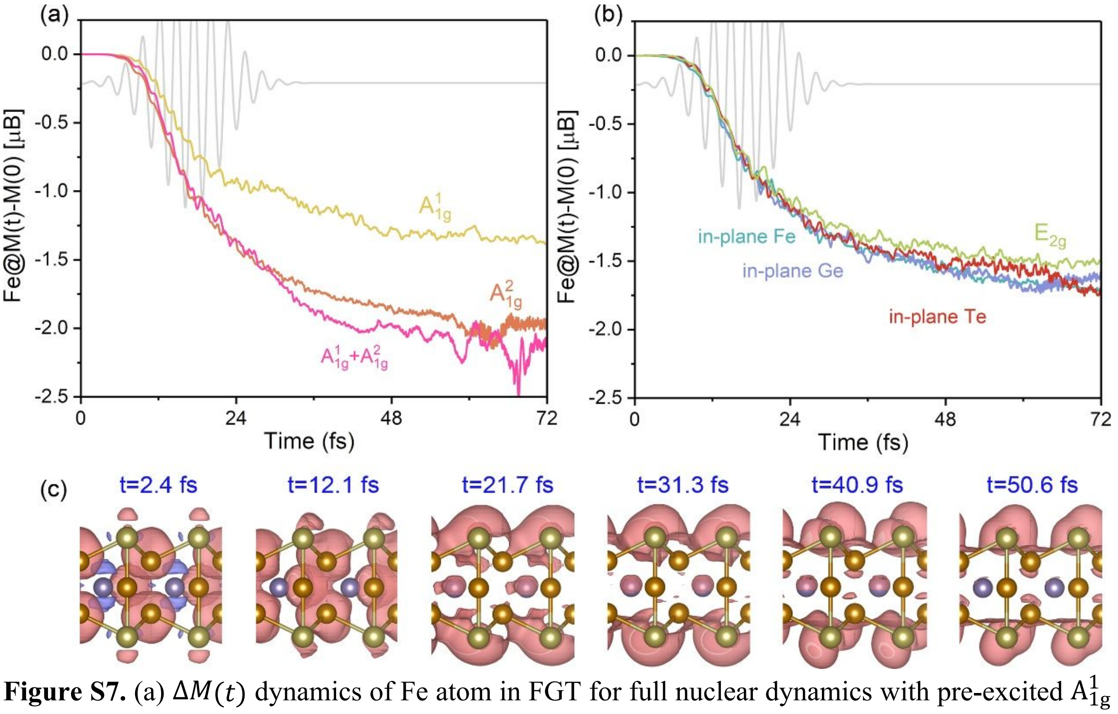

Figure S7. (a) $\Delta M(t)$ dynamics of Fe atom in FGT for full nuclear dynamics with pre-excited A_{1g}^1 and A_{1g}^2 coherent phonons and when two out-of-plane modes are excited together. (b) The same results of in-plane coherent phonons including E_{2g}, in-plane Fe, in-plane Ge and in-plane Te modes. (c) Snapshots of the magnetization density of FGT with pre-excited A_{1g}^2 coherent phonon for full nuclear dynamics at different time points. Red (purple) domains of the iso-surface represent spin-up (spin-down) electrons. The iso-surface is set as 0.002 e/Å^3.

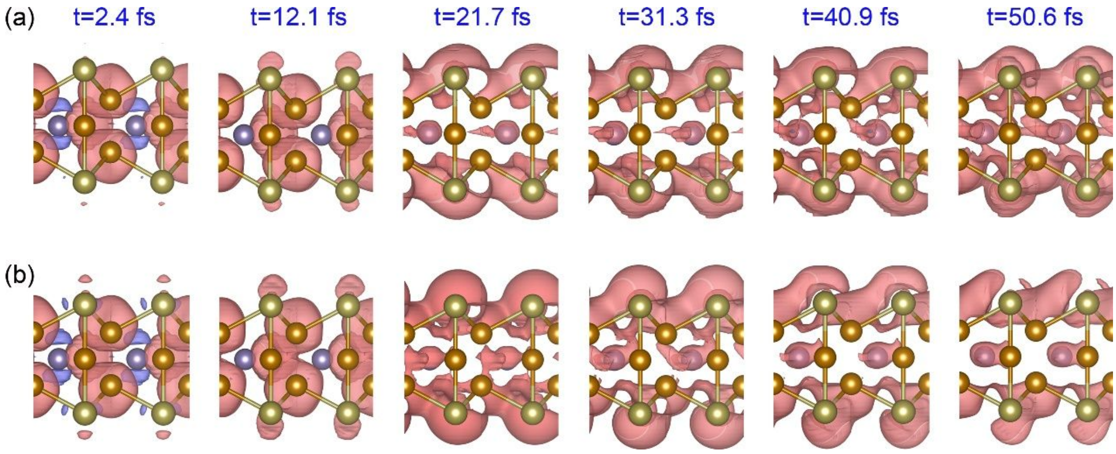

Figure S8. Snapshots of the magnetization density of FGT for full nuclear dynamics with pre-excited (a) A_{1g}^{1} and (b) E_{2g} coherent phonon at different time points. Red (purple) domains of the iso-surface represent spin-up (spin-down) electrons. The iso-surface is set as 0.002 e/Å^3.

Fluence density and phonon amplitude-dependent spin moment loss

We examine the A_{1g}^{2} coherent phonon as a case and investigate the spin moment dynamics of Fe atoms in FGT under the influence of laser pulses with different pulse fluence, as shown in Figure S9a. The results reveal that while the spin moment loss exhibits low sensitivity to pulse fluence, a relative dependence is still evident. First, the spin moment loss experiences a slight increase with rising pulse fluence before 24 fs (inset panel in Figure S9a), attributed to the OISTR phenomenon. Following the dissipation of the laser pulse after 24 fs, the spin moment loss shows the opposite trend compared to the earlier time scale. This behavior can be understood as the Fe atom nuclei exhibiting larger adiabatic displacements towards equilibrium with increasing pulse fluences. As a result, weaker interactions between nuclei and electronic systems occur, leading to a reduction in spin moment loss over longer time scales. This suggests that if the fluence threshold is sufficiently strong to restore the Fe atom nuclei from their pre-excited positions to equilibrium, the loss of spin moment will resemble scenarios where nuclear dynamics are not considered. Besides, we investigate the impact of phonon amplitude on magnetization dynamics as well. Figure S9b illustrates that there is a negligible difference in the amount of spin moment loss when the amplitude of the phonon varies from 0.008 nm to 0.020 nm. Hence, we suggest that the selection of the initial pulse based on the double-pump technique should solely consider the type of coherent phonons targeted for pre-excitation, without regard to the intensity of the excited coherent phonons.

Note that Sharma et al. have reported that the coupling phonon excitations of the nuclear to spin and charge can lead to femto-phono-magnetism in FePt system, revealing the significant effect of nuclear dynamics on controlling spin moment loss at ultrafast timescale.[19] In our work, we verify that such significant phenomena of coherent phonon excitation induced additional spin moment loss in 2D ferromagnet monolayer. Beyond this, the spin moment loss over longer timescales and the effects of stronger phonon amplitudes are also explored using frozen phonon NAMD simulations. This approach fully reveals the phonon-dependent spin dynamics processes ranging from

femtoseconds to picoseconds.

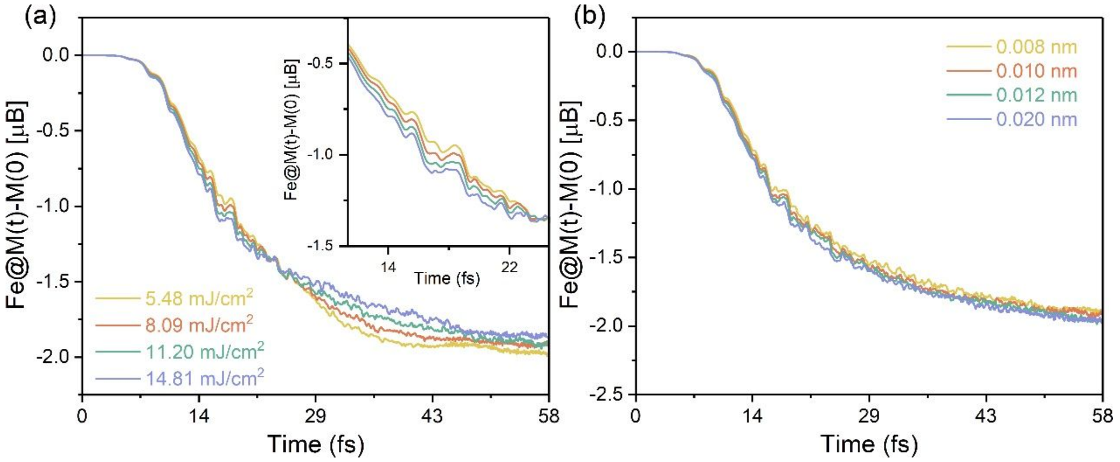

Figure S9. $\Delta M(t)$ dynamics of Fe atom in FGT for full nuclear dynamics with (a) different fluence (FWHM=12.21 fs) and (b) different amplitudes of pre-excited A_{1g}^2 phonon.

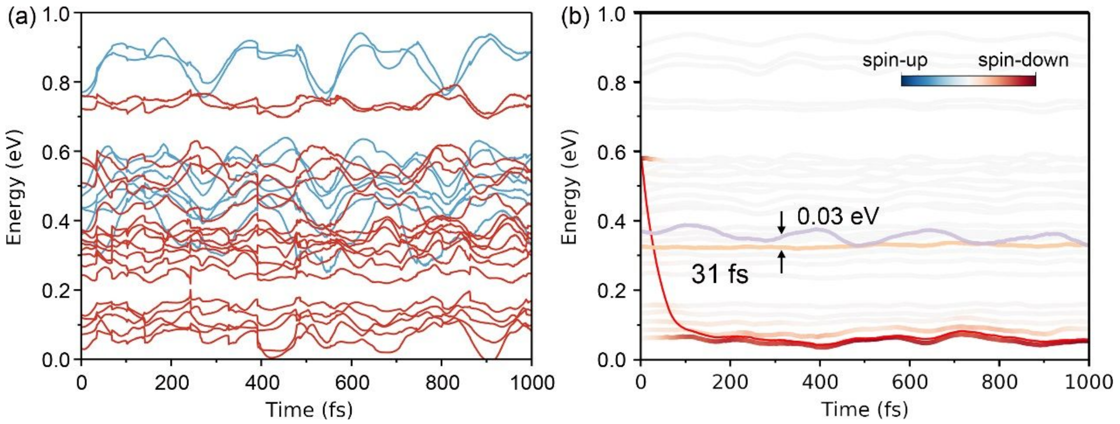

Figure S10. (a) Time evolution of the spin-resolved energy states and (b) energy relaxation of the excited spin-down electron of FGT at the Γ point without excitation of coherent phonon. The color map indicates orbital localization. The time data are fitted using the exponential function $f(t) = a + b\exp(-t/\tau)$. The lowest spin-up state and the adjacent spin-down state is marked as purple and yellow line respectively. The average energy gap is 0.03 eV, much lower than the 0.19 eV, 0.17 eV and 0.10 eV for A_{1g}^1, A_{1g}^2 and E_{2g} respectively in Figure 4.

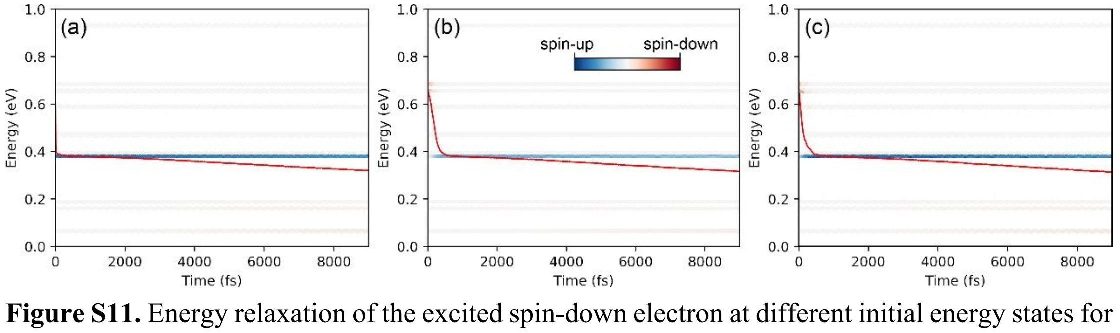

Figure S11. Energy relaxation of the excited spin-down electron at different initial energy states for excited A_{1g}^1 mode. The color map indicates orbital localization.

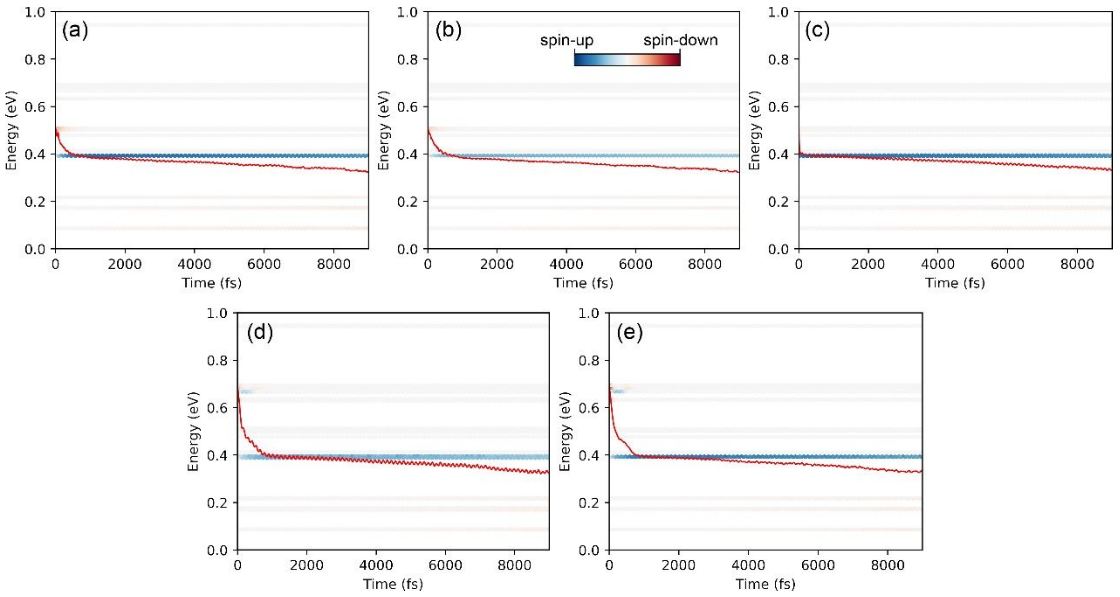

Figure S12. Energy relaxation of the excited spin-down electron at different initial energy states for excited A_{1g}^2 mode. The color map indicates orbital localization.

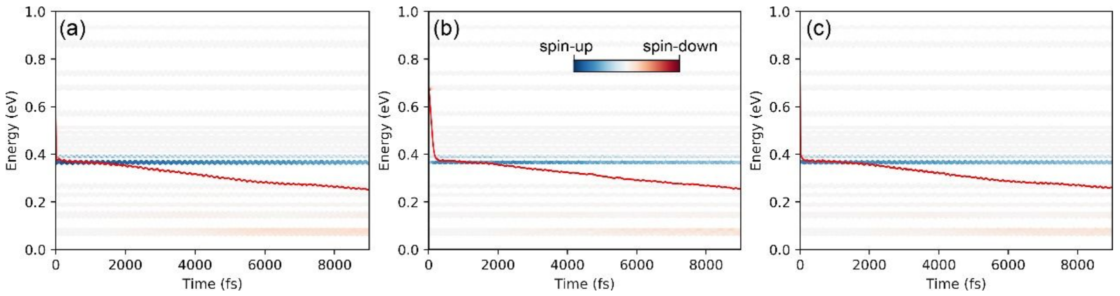

Figure S13. Energy relaxation of the excited spin-down electron at different initial energy states for excited E_{2g} mode. The color map indicates orbital localization.

REFERENCES

(1) Runge, E.; Gross, E. K. U. Density-Functional Theory for Time-Dependent Systems. *Phys. Rev. Lett.* **1984**, *52*, 997–1000.

(2) Yu, R.; Singh, D.; Krakauer, H. All-Electron and Pseudopotential Force Calculations Using the Linearized-Augmented-Plane-Wave Method. *Phys. Rev. B* **1991**, *43*, 6411–6422.

(3) Kresse, G.; Furthmuller, J. Efficient Iterative Schemes for Ab Initio Total-Energy Calculations Using a Plane-Wave Basis Set. *Phys. Rev. B* **1996**, *54*, 11169–11186.

(4) Kresse, G.; Hafner, J. Ab Initio Molecular-Dynamics Simulation of the Liquid-Metal--Amorphous-Semiconductor Transition in Germanium. *Phys. Rev. B* **1994**, *49*, 14251–14269.

(5) Perdew, J. P.; Burke, K.; Ernzerhof, M. Generalized Gradient Approximation Made Simple. *Phys. Rev. Lett.* **1996**, *77*, 3865–3868.

(6) Kresse, G.; Joubert, D. From Ultrasoft Pseudopotentials to the Projector Augmented-Wave Method. *Phys. Rev. B* **1999**, *59*, 1758–1775.

(7) Togo, A.; Tanaka, I. First Principles Phonon Calculations in Materials Science. *Scr. Mater.*

2015, *108*, 1–5.

(8) Dewhurst, J. K.; Sharma, S. Elk Code. *elk.sourceforge.net*.

(9) Zheng, Z.; Zheng, Q.; Zhao, J. Spin-Orbit Coupling Induced Demagnetization in Ni: Ab Initio Nonadiabatic Molecular Dynamics Perspective. *Phys. Rev. B* **2022**, *105*, 085142.

(10) Zheng, Q.; Chu, W.; Zhao, C.; Zhang, L.; Guo, H.; Wang, Y.; Jiang, X.; Zhao, J. Ab Initio Nonadiabatic Molecular Dynamics Investigations on the Excited Carriers in Condensed Matter Systems. *WIREs Comput. Mol. Sci.* **2019**, *9*, e1411.

(11) Akimov, A. V.; Prezhdo, O. V. The PYXAID Program for Non-Adiabatic Molecular Dynamics in Condensed Matter Systems. *J. Chem. Theory Comput.* **2013**, 9, 4959−4972.

(12) Zhang, L.; Zheng, Q.; Xie, Y.; Lan, Z.; Prezhdo, O. V.; Saidi, W. A.; Zhao, J. Delocalized Impurity Phonon Induced Electron-Hole Recombination in Doped Semiconductors. *Nano Lett.* **2018**, *18*, 1592–1599.

(13) Deng, Y.; Yu, Y.; Song, Y.; Zhang, J.; Wang, N. Z.; Sun, Z.; Yi, Y.; Wu, Y. Z.; Wu, S.; Zhu, J.; Wang, J.; Chen, X. H.; Zhang, Y. Gate-Tunable Room-Temperature Ferromagnetism in Two-Dimensional Fe_3GeTe_2. *Nature* **2018**, *563*, 94–99.

(14) Fei, Z.; Huang, B.; Malinowski, P.; Wang, W.; Song, T.; Sanchez, J.; Yao, W.; Xiao, D.; Zhu, X.; May, A. F.; Wu, W.; Cobden, D. H.; Chu, J.-H.; Xu, X. Two-Dimensional Itinerant Ferromagnetism in Atomically Thin Fe_3GeTe_2. *Nat. Mater.* **2018**, *17*, 778–782.

(15) Yin, T.; Ulman, K. A.; Liu, S.; Granados Del Águila, A.; Huang, Y.; Zhang, L.; Serra, M.; Sedmidubsky, D.; Sofer, Z.; Quek, S. Y.; Xiong, Q. Chiral Phonons and Giant Magneto-Optical Effect in $CrBr_3$ 2D Magnet. *Adv. Mater.* **2021**, *33*, 2101618.

(16) Luo, J.; Lin, T.; Zhang, J.; Chen, X.; Blackert, E. R.; Xu, R.; Yakobson, B. I.; Zhu, H. Large Effective Magnetic Fields from Chiral Phonons in Rare-Earth Halides. *Science* **2023**, *382*, 698–702.

(17) Fransson, J. Chiral Phonon Induced Spin Polarization. *Phys. Rev. Res.* **2023**, *5*, L022039.

(18) Bonini, J.; Ren, S.; Vanderbilt, D.; Stengel, M.; Dreyer, C. E.; Coh, S. Frequency Splitting of Chiral Phonons from Broken Time-Reversal Symmetry in CrI_3. *Phys. Rev. Lett.* **2023**, *130*, 086701.

(19) Sharma, S.; Shallcross, S.; Elliott, P.; Dewhurst, J. K. Making a Case for Femto-Phono-Magnetism with FePt. *Sci. Adv.* **2022**, *8*, eabq2021.
